# Factors associated with the risk of violence against older adult women: a cross-sectional study

**DOI:** 10.1590/1518-8345.4039.3394

**Published:** 2021-01-08

**Authors:** Rute Costa Régis De Sousa, Gleicy Karine Nascimento De Araújo, Rafaella Queiroga Souto, Renata Clemente Dos Santos, Rafael Da Costa Santos, Luana Rodrigues de Almeida

**Affiliations:** 1 Universidade Federal de Pernambuco, Recife, PE, Brazil.; 2 Universidade Federal da Paraíba, João Pessoa, PB, Brazil.; 3 Universidade Federal da Paraíba, Departamento de Enfermagem em Saúde Coletiva, João Pessoa, PB, Brazil.; 4 Universidade Federal da Paraíba, Escola de Enfermagem, João Pessoa, PB, Brazil.

**Keywords:** Forensic Nursing, Exposure to Violence, Geriatric Nursing, Women, Public Health, Aged, Enfermagem Forense, Exposição à Violência, Enfermagem Geriátrica, Mulheres, Saúde Pública, Idoso, Enfermagem Forense, Exposição à Violência, Enfermagem Geriátrica, Mulheres, Saúde Pública, Idoso

## Abstract

**Objective:**

to identify the factors associated with the risk of violence against older adult women.

**Method:**

this is a quantitative, analytical, and cross-sectional research conducted with 122 older adult females in the city of Recife, state of Pernambuco, Brazil. Data collection was carried out using validated instruments adapted to Brazil. The analysis was performed using descriptive statistics (absolute and relative frequency) and inferential statistics (Pearson’s chi- square, Spearman’s correlation test, and Multiple Logistic Regression).

**Results:**

there was prevalence of a risk of abuse against older adult women under 70 years of age, literate, without a stable relationship, living alone, without any work activity, and who had an income higher than the minimum wage. There is a significant association between the risk of violence among older women with a higher number of chronic health conditions (24; 77.4%), and who are less active in advanced activities (42; 70.0%). A reduction in quality of life and satisfaction with life, and the onset of depressive symptoms, increase the risk of violence.

**Conclusion:**

multimorbidity, low functional capacity, depressive symptoms, low quality of life and low satisfaction with life, a high number of chronic conditions, depressive symptoms, and functional dependence to perform daily activities can be conditioning factors for the emergence of abuse against older adults.

## Abstract (pt)

**Objetivo:**

identificar os fatores associados ao risco de violência contra mulheres idosas.

**Método:**

trata-se de uma pesquisa quantitativa, analítica e transversal, realizada com 122 mulheres idosas na cidade de Recife, Estado de Pernambuco, Brasil. A coleta de dados foi realizada por meio de instrumentos validados e adaptados para o Brasil. A análise foi realizada por meio de estatística descritiva (frequência absoluta e relativa) e estatística inferencial (qui-quadrado de Pearson, teste de correlação de Spearman e regressão logística múltipla).

**Resultados:**

houve prevalência de risco de maus-tratos em mulheres idosas com menos de 70 anos, alfabetizadas, sem união estável, morando sozinhas, sem nenhuma atividade laboral e com renda superior a um salário mínimo. Existe uma associação significativa entre o risco de violência entre mulheres idosas com maior número de doenças crônicas (24; 77,4%) e menos ativas em atividades avançadas (42; 70,0%). A redução da qualidade de vida e do nível de satisfação com a vida e o aparecimento de sintomas depressivos aumentam o risco de violência.

**Conclusão:**

multimorbidade, baixa capacidade funcional, sintomas depressivos, baixa qualidade de vida e baixa satisfação com a vida, elevado número de condições crônicas, sintomas depressivos e dependência funcional para a realização de atividades diárias podem ser fatores condicionantes para o surgimento de maus-tratos contra idosos.

## Abstract (es)

**Objetivo:**

identificar los factores asociados con el riesgo de violencia contra mujeres de edad avanzada.

**Método:**

se trata de una investigación cuantitativa, analítica y transversal realizada con 122 mujeres de edad avanzada en la ciudad de Recife, estado de Pernambuco, Brasil. La recopilación de datos se llevó a cabo utilizando instrumentos validados y adaptados al Brasil. En el análisis se utilizaron estadísticas descriptivas (frecuencia absoluta y relativa) y estadísticas inferenciales (prueba chi-cuadrado de Pearson, prueba de correlación de Spearman y Regresión Logística Múltiple).

**Resultados:**

se observó prevalencia de riesgo de abuso contra mujeres mayores de 70 años, alfabetizadas, sin una relación estable, que viven solas, sin ninguna actividad laboral y que tienen un ingreso superior al salario mínimo. Existe una asociación significativa entre el riesgo de violencia entre las mujeres de edad avanzada con un mayor número de condiciones de salud crónicas (24; 77,4%), y aquellas con menos actividad (42; 70,0%). La reducción de la calidad de vida y la insatisfacción con la vida, así como la aparición de síntomas de depresión, aumentan el riesgo de violencia.

**Conclusión:**

la multimorbilidad, la baja capacidad funcional, los síntomas de depresión, la baja calidad de vida, la insatisfacción con la vida, un elevado número de afecciones crónicas y la dependencia funcional para realizar actividades cotidianas pueden ser factores condicionantes para la aparición del abuso contra personas de edad avanzada.

## Introduction

The increase in the number of dependent older adults can be related to the ageing process, which often comes with physical, emotional, and cognitive limitations. When associated with low social status, family unpreparedness for care provision, and a recurrent history of intra-family conflicts, such problems can all be factors in the occurrence of the phenomenon of abuse against older adults(1).

Abuse against older adults is defined as any type of action or omission that occurs individually or collectively, on a single occasion or repetitively, within a relationship where trust or expectation exists, causing harm and/or distress to the older adult^(^ 2 ^)^. The discussion about the phenomenon still has little visibility in the academic community^(^ 3 ^)^; however, it deserves special attention given the vulnerability and the harms resulting from this phenomenon to public health and to the quality of life of the older population^(^ 4 ^)^.

Despite being listed in the literature, the presence of dementia, advanced age, dependence to perform basic living activities, unfavorable socioeconomic conditions, and female gender are risk factors for the older adults to be victims of violence^(^ 5 ^)^. Violence Against Older Adults (VAOA) does not occur uniformly, and it is usually found in more than one modality. For example, a study conducted in Minas Gerais, Brazil, showed a prevalence of physical and psychological violence, followed by neglect, financial violence, torture, and sexual violence^(^ 6 ^)^. Older women are more likely to be victims of violence, which is predominantly perpetrated in the home and family environment^(^ 7 ^)^. Such evidence has frequently been observed in different studies and contexts(4,8-9), which leads us to consider that women are more exposed to experiencing situations of violence and, thus, that gender is a risk factor for the phenomenon. This condition of vulnerability is explained by gender inequalities, whereby women are subjugated and oppressed at all ages, and this increases in later life, when power relations involving other aggressive elements than gender can be found.

In a survey conducted with 7,257 women belonging to different age groups, 65.1% of those who were over 65 years old reported having suffered physical or sexual violence, compared to 8% of women between 16 and 49 years old, and 3% of women aged between 50 and 65 years old^(^ 10 ^)^.

In order to deal with this phenomenon, it is necessary to work with trained professionals to properly assist the older victims of violence. The area of Forensic Nursing (FN) was regulated in Brazil in 2011. It is a specialty that acts in situations of violence, such as screening and assisting the victims^(^ 11 ^)^.

A methodological study, developed with the objective of determining the skills destined to FN to care for older adults in violent situations, points out in its results that there are 47 general skills for exercise, that includes identifying confirmed and/or suspected cases of abuse against older adults, reporting cases of violence, and implementing intervention plans on victims, aggressors, and/or their families^(^ 12 ^)^.

The scope of FN in the area of abuse, sexual abuse, trauma, and other forms of violence focuses on establishing human responses in all contexts and life cycles, including the older adults, as well as developing, promoting, monitoring, and implementing responses to the harms that occur as a result of violence through the application of care practices^(^ 11 ^)^. In order to strengthen violence prevention actions, the identification of factors that influence the occurrence of violence is essential in planning an effective and resolving action^(^ 13 ^)^.

Considering the problem raised, the development of studies that understand the associated factors and abuse against older adults among individuals who live in the community is justified, in order to give greater visibility to this phenomenon and to develop an FN view of the practice among the registered nurses, enabling the proposal of possible interventions and measures to prevent abuse against older adults.

For the subject matter under discussion, the following question can be asked: What are the factors that influence the risk of abuse against older adult women? Therefore, the present study aims to identify the factors associated with the risk of violence against older adult women.

## Method

This is a quantitative, descriptive, and cross-sectional study guided by the STROBE (Strengthening the Reporting of Observational Studies in Epidemiology) tool, which was developed to evaluate the quality of observational studies (case-control, cohort, and cross-sectional)^(^ 14 ^)^, carried out from 2016 to 2017 in the city of Recife, state of Pernambuco, Brazil.

The study included all the older adults registered in the area covered by three family health teams from the Family Health Unit, located in the III Micro-area of the IV Health District, Recife, Pernambuco. The population consisted of 1,209 individuals.

The sample was calculated according to the finite population formula for epidemiological studies. With this calculation, the resulting sample consisted of 159 older adults. For evaluation purposes, only data referring to female participants were used since this is a population more vulnerable to violence, as pointed out in the literature^(^ 5 ^)^, which included 122 individuals.

Systematic random sampling was used, and the number of participants was determined proportionally among the three teams of the health unit. Out of every five older adults on each team’s list, one was chosen and invited to take part in the survey.

The study included individuals aged 60 or over, and excluded those who had severe hearing (low hearing acuity or deafness) or visual (low visual acuity or blindness) impairments. The respective exclusion criteria were detected by the researcher through observation or information from those responsible for them. A total of 17 individuals were excluded due to these criteria.

The place of data collection was the home of the older adult, after explanation of the research objectives and data confidentiality. Subsequently, the individual who was available and had interest in participating signed the Free and Informed Consent Form (FICF). The participants received no financial incentive to participate in the survey.

The interviewer was accompanied by the Community Health Agent to the home of the older adult in order to provide maximum safety, as the agent is a member of the health team and knows the community.

In order that the participants’ schooling level did not prevent them from reading and answering the questions, the researcher read aloud all the questions from the questionnaires. In addition, the interviewer asked to conduct the survey only with the interviewee, in a private space, thereby building a more favorable environment for the older adult to feel safe in answering questions which were mainly related to violence.

Data collection lasted approximately 40 minutes. No losses were observed in the development of the research since the researcher clarified that collection could be interrupted and resumed at other times.

The following validated instruments adapted to Brazil were used for data collection: the Brazil Old Age Schedule (BOAS)^(^ 15 ^)^; the Hwalek-Sengstock Elder Abuse Screening Test (H-S/EAST)^(^ 16 ^)^; the Self-Reported Chronic Conditions questionnaire^(^ 17 ^)^; the Mini Mental State Examination (MMSE)^(^ 18 ^)^; Advanced Activities of Daily Living (AADLs)^(^ 19 ^)^; Instrumental Activities of Daily Living (IADLs)^(^ 20 ^)^; Basic Activities of Daily Living (BADLs)^(^ 21 ^)^; and the World Health Organization Quality of Life (WHOQOL-OLD) module^(^ 22 ^)^.

The sociodemographic characterization of the assessed population was performed using the BOAS instrument, which is divided into sections that include general information, physical health, and use of medical, dental, and mental health services^(^ 15 ^)^. Questions were extracted from this instrument referring to age, marital status, literacy, housing arrangements, work, and income (6 questions).

The H-S/EAST is an American instrument, consisting of 15 questions that analyze the risk of abuse against older adults. One point is given for each affirmative answer, except for items 1, 6, 12, and 14, where the point is given for negative answers. A score of three or more indicates an increased risk of a type of violence present^(^ 16 ^)^.

The Self-Reported Chronic Conditions questionnaire assesses, in 7 questions, the presence of chronic pathologies, namely: angina or infarction, stroke or cerebrovascular accident (CVA), cancer, arthritis or rheumatism, lung disease, depression, and osteoporosis^(^ 17 ^)^. The MMSE was applied to assess cognitive impairment among the older adults by means of 30 questions. The score of this instrument can range from 0 to 30 points, where the cutoff can vary according to the interviewee’s schooling, being at 13 points for illiteracy, 18 for individuals with a low or medium schooling level, and 26 for those with a high level of schooling^(^ 18 ^)^.

The participants’ functional capacity was assessed according to the AADLs, IADLs and BADLs. The AADLs are 12 adapted questions that refer to the participation of the older adults in more complex activities such as educational, civic, religious, and leisure activities, offering three possible answers: never done, stopped doing, and still does. The individuals who perform only three or fewer activities are classified as less active^(^ 19 ^)^. The IADLs are 7 questions about activities of medium complexity such as telephone use, use of transportation, performing purchases, and money management^(^ 20 ^)^, while the BADLs were based on the Katz index, verifying functions such as moving around, hygiene, feeding, and sphincter control in 6 questions. All the scales classified the older adults as independent or dependent based on the need for help or the inability to perform any of the activities evaluated^(^ 21 ^)^.

The WHOQOL-OLD module assesses quality of life. It consists of 24 questions and the answers are indicated on a Likert scale (from 1 to 5) attributed to six elements, namely: sensory functioning; autonomy; past, present and future activities; social participation; death and dying; and intimacy^(^ 22 ^)^.

The risk of violence was the dependent variable. The independent variables were the following: age, marital status, literacy, payed work, income, living arrangement, chronic conditions, activities of daily living, quality of life, and cognitive impairment.

The collected data were inserted in the double entry modality by independent typists in the Statistical Package for the Social Sciences (SPSS), version 21.0, and the disagreements were reviewed and corrected by the data collection coordinator.

Subsequently, data analysis was performed using descriptive (absolute and relative frequency) and inferential (Pearson chi-square, Spearman correlation test, and Multiple Logistic Regression) statistics. The non-parametric test was established because, according to the result of the Kolmogorov-Smirnov normality test, the variables did not present normal distribution. A significance level of 5% (p<0.05) was adopted for all the analyses.

The entry criteria of the variables in the logistic regression model was set at p<0.02 based on the result of the association test. In the end, a value of 0.05 or less was considered significant.

This research was submitted to the Ethics and Research Committee of the Federal University of Pernambuco for consideration and was approved under Opinion No. 1413599/16. All the recommendations and ethical principles foresaw in research involving human beings have been respected and followed in accordance with Resolution 466/12, established by the Brazilian Health Council.

## Results

In the sample there was prevalence of women with a maximum age of 70 years old (72; 59.0%); literate (85; 69.7%); single (92; 75.4%); living with someone (106; 86.9%); not working (103; 88.0%); and with an income of up to 1 minimum wage (94; 77.0%). The risk of violence was present in 59.0% (n=72) of the interviewees.

Table 1 below shows the data regarding the association between these characteristics and the risk of violence. No statistically significant association was observed between the variables; however, there was prevalence of the risk of violence for older women over 70 years of age, those who were literate, without a stable relationship, living alone, those who had no work activity, and those with an income greater than one minimum wage.

**Table 1:** Risk of violence as a function of the sociodemographic characteristics of the older adult women taking part in the survey. Recife, PE, Brazil, 2016-2017, N=122

Variables	Risk of violence		p-value*
	With risk	Without risk	
Age	n (%)	n (%)	
≤70 years old	42 (58.3)	30 (41.7)	0.85
>70 years old	30 (60.0)	20 (40.0)	
Literate			
Yes	51 (60.0)	34 (40.0)	0.73
No	21 (56.8)	16 (43.2)	
Marital status			
Married/Living together	17 (56.7)	13 (43.3)	0.76
Widowed/Separated/ Never married	55 (59.8)	37 (40.2)	
Living arrangement			
Lives alone	10 (62.5)	6 (37.5)	0.76
Lives with someone	62 (58.5)	44 (41.5)	
Working now?			
Yes	6 (42.9)	8 (57.1)	0.19
No	63 (61.2)	40 (38.8)	
Monthly income			
Up to 1 minimum wage	54 (57.4)	40 (42.6)	0.51
More than 1 minimum wage	18 (64.3)	10 (35.7)	

* Pearson's chi-square test

Table 2 presents the risk of violence according to certain factors such as the number of self-reported chronic conditions, and the advanced, instrumental, and basic activities of daily living. There is a significant association between the risk of violence against older women and the higher number of chronic conditions (24; 77.4%), and less activity (42; 70.0%), according to the AADL scale. The crossing of the risk of violence and the IADL and BADL scales was not significant; however, dependence was predominant among older adult women at risk of violence.

**Table 2:** Association of the risk of violence and the factors evaluated among the participants of the survey. Recife, PE, Brazil, 2016-2017, N=122

Variables	Risk of violence		p-value*
	With risk	Without risk	
	n (%)	n (%)	
No. of self-reported chronic conditions			
0 to 3	48 (52.7)	43 (47.3)	0.01
4 to 7	24 (77.4)	7 (22.6)	
AADLs^†^			
More active	30 (48.4)	32 (51.6)	0.01
Less active	42 (70.0)	18 (30.0)	
IADLs^‡^			
Independent	30 (53.6)	26 (46.4)	0.21
Dependent	42 (64.6)	23 (35.4)	
BADLs^§^			
Independent	55 (57.3)	41 (42.7)	0.33
Dependent	17 (68.0)	8 (32.0)	

* Pearson's chi-square test;

† AADL = Advanced activities of daily living;

‡ IADL = Instrumental activities of daily living;

§ BADL = Basic activities of daily living

Table 3 shows the multiple logistic regression model corresponding to the risk of violence. Only variables with p<0.02 in the bivariate analyses were included in the model. The data allow us to infer that older women with 4 to 7 chronic conditions who are less active are 2.69 and 2.22 times more likely to develop some risk of violence, respectively.

**Table 3:** Variables associated to the risk of violence through adjusted logistic regression. Recife, PE, Brazil, 2016-2017, N=122

Variables	OR*	CI^†^	p-value^‡^
No. of self-reported conditions			
0 to 3	1.00	-	-
4 to 7	2.69	[1.03-7.01]	0.04
Advanced daily living activities			
More active	1.00	-	-
Less active	2.22	[1.03-4.75]	0.04

Adjusted R²: 0.111; Accuracy of the test: 0.336;

* OR = Odds ratio;

† CI = Confidence interval;

‡ Significance of the test.

Table 4 shows the result of the correlation analysis between the total scores of the H-S/EAST, MMSE, and WHOQOL instruments. Through the negative correlation, it can be observed that, as quality of life decreases, the older adults are at a higher risk of violence.

**Table 4:** Correlation between the total score of the H-S/EAST and the total scores of MEEM and WHOQOL. Recife, PE, Brazil, 2016-2017, N=122

Variables	Total score of H-S/EAST*	
	Correlation coefficient	p-value^†^
Total score of MMSE^‡^	0.008	0.934
Total score of WHOQOL^§^	-0.253	0.007

* H-S/EAST = Hwalek-Sengstock Elder Abuse Screening Test;

† Spearman Correlation Test;

‡ MMSE = Mini Mental State Examination;

§ WHOQOL = World Health Organization Quality of Life module

## Discussion

Considering the predictive factors as a guide for the professional practice enables the professional to turn care to the real needs of the study population. When referring to VAOA, FN aims to improve the assistance to cases considered forensic by presenting specific skills to face this phenomenon^(^ 23 ^)^. The sample characterization data indicates the situations of predominance for the risk of violence among older adults, which need a view of Nursing from the forensic perspective.

The participants who attained the risk score for abuse were predominantly over the age of 70; this data corroborates those found in some studies in the literature(24-25), such as a study conducted in Iran in which abuse is present in 93% (n=57) of the women over 72 years old^(^ 26 ^)^. Women in this age group can be at greater risk of violence due to the increased vulnerability that accompanies advancing age^(^ 24 ^)^.

The results showed that literacy was characterized as a risk factor for violence in older adult women, diverging from the literature, which states that older adults with low schooling levels are more likely to suffer abuse^(^ 27 ^)^. However, another study asserted that the risk of sexual or physical violence increases as the years of study increase^(^ 28 ^)^. The prevalence of risk among the older adults can be justified by the better perception of violence among those with a higher schooling level.

Despite not being statistically significant, marital status is also a risk factor when it comes to violence. A systematic review recorded that not being in a relationship is a factor associated with violence in older adults^(^ 29 ^)^, and another study shows this same marital profile in women with a higher prevalence of physical and sexual violence^(^ 30 ^)^.

It is possible to verify that the risk was more prevalent among those who earned more than one minimum wage. Another study conducted with older Chinese women living in the United States found similar results, i.e., that abuse was more prevalent among individuals with higher income levels^(^ 31 ^)^. These findings seem contradictory since older adult women who have financial difficulties are 2.5 times more likely to suffer more severe abuse^(^ 32 ^)^. Nonetheless, considering that many older adults contribute significantly to the family income, this leads to older women with greater economic power to be at a greater risk of abuse, which can be perpetrated by the family members themselves through the appropriation of the money or property belonging to this older adult woman^(^ 33 ^)^.

An association (p=0.01) was found between the number of self-reported conditions and the risk of violence and, in logistic regression, older adult women who reported the highest number of conditions, i.e., the greatest multimorbidity, were 2.69 times more likely to be at risk of abuse (CI=95%, 1.03-7.01). The longer life expectancy of women compared to men in Brazil^(^ 34 ^)^ and worldwide^(^ 35 ^)^ has led to a feminization of old age^(^ 36 ^)^, without this automatically translating into healthy aging, leading to a high prevalence of multimorbidity in older women and older people in general^(^ 37 ^-^ 39 ^)^.

Multimorbidity is the occurrence of two or more chronic conditions in the same individual^(^ 40 ^)^, and it is associated with elements such as loss of functional capacity^(^ 39 ^,^ 41 ^)^, decreased grip strength^(^ 40 ^)^, frailty^(^ 42 ^)^, and cognitive impairment^(^ 43 ^)^, all of which are also associated with abuse against older adults. In addition, the literature shows that older adults with a high number of comorbidities have a higher chance of suffering violence^(^ 44 ^)^. Reinforcing this analysis, a study developed in Dakahlia, Egypt, with 272 older adults concluded, based on the regression analysis, that having no chronic comorbidities is a protective factor for older adult women^(^ 45 ^)^.

The decrease in functional capacity potentially induced by the presence of morbidities associated with the risk of violence is a very evident fact when we analyze Table 2, where it is observed that the older adult women who are less active in AADLs had higher prevalence (70.0%, n=42) of risk of violence (p=0.01); and Table 3, which shows that older adults who are less active in AADLs are 2.22 times more likely to be at risk of abuse (CI=95%, 1.03-4.75).

FN is responsible for acting in the prevention of VAOA, as well as for planning actions that prevent or minimize functional decline, which is recognized as a measure that can reduce the risk of violence among this population^(^ 46 ^-^ 47 ^)^. The literature indicates that the functional dependence of the older adult can increase exposure to violence by 2.20 times, as it increases the dependence relationship between the older victims and the likely aggressor^(^ 48 ^)^.

A research study developed in Rio de Janeiro with dependent older adults sought to list the factors associated with violence and, in the findings from the association, they observed a higher prevalence of violence among older adults with cognitive impairment^(^ 49 ^)^. There are few notes in the literature that show the correlation between the risk of violence and the cognitive impairment that was observed in the results of the present study, so that it is not possible to determine the relationship between cause and effect for both outcomes. A high cognitive deficit can increase the risk of violence, as well as the risk of violence can worsen the cognitive function of older adult women^(^ 50 ^)^.

Another variable that also correlated with the risk of abuse was the WHOQOL total score: the correlation coefficient was negative, thus pointing to an inversely proportional relationship between the two variables, i.e., the higher the WHOQOL score (higher quality of life), the lower the HS-EAST score (lower risk of abuse), or vice versa.

A study conducted with older adults in China found that self-neglect is a risk factor for deficient quality of life^(^ 51 ^)^, and that other factors also associated with quality of life^(^ 52 ^)^ are years of study, self-rated health, number of chronic conditions, and physical activity^(^ 53 ^)^.

Quality of life is also associated with the prejudice that the older adults have about themselves and about aging, so FN is responsible for promoting positive attitudes towards old age and the self-perception of older adult women, thus leading to an improvement in quality of life and, consequently, to a reduced likelihood of abuse^(^ 54 ^-^ 55 ^)^.

The results support the focus of the performance of FN for the study population, pointing out that functional capacity, cognitive decline, and quality of life, are factors that increase the risk of violence among older adults, so any intervention in these aspects can be satisfactory in preventing violence. In addition, these factors should attract the attention of forensic nurses in screening older adults who are victims of violence^(^ 13 ^)^.

The study limitations include the absence of validated instruments for the Brazilian scenario that identify all types of violence, restricting the deepening of the discussion on this phenomenon. In addition, there is a lack of studies that relate FN and its exercise in the context of VAOA, thus preventing a clear understanding of the scientific Nursing community about this field of action.

## Conclusion

It can be concluded that factors such as multimorbidity, low functional capacity, depressive symptoms, low quality of life and low satisfaction with life, depressive symptoms, and functional dependence for daily activities, are associated with the risk of violence against older adult women.

The study contributes to the academic field and to the Nursing practice since it provides subsidies for decision-making based on proven evidence, which demonstrates the predictive factors of violence against older adult women. In addition, it also to strengthens the field of work of FN, as well as conducting new research studies of situational diagnosis and proposing interventions to combat the phenomenon of violence.
